# The development and mixed-method stakeholder evaluation of a sport-related concussion symptom and treatment monitoring system in community rugby

**DOI:** 10.1007/s11845-026-04485-x

**Published:** 2026-06-19

**Authors:** Laura C. Power, Ian C. Kenny, John J.E. Mulvihill, Kilian L. Bibby, Helen Purtill, Aaron J. Zynda, Michael W. Collins, Anthony P. Kontos, Tom M. Comyns

**Affiliations:** 1 Department of Physical Education and Sport Sciences, University of Limerick, Limerick, Ireland; 2 Health Research Institute, University of Limerick, Limerick, Ireland; 3 Sport & Human Performance Research Centre, University of Limerick, Limerick, Ireland; 4 Lero, the Research Ireland Centre for Software, Limerick, Ireland; 5 School of Engineering and Bernal Institute, University of Limerick, Limerick, Ireland; 6 Department of Mathematics & Statistics, University of Limerick, Limerick, Ireland; 7 Department of Orthopaedic Surgery, University of Pittsburgh Medical Center Sports Medicine Concussion Program, University of Pittsburgh School of Medicine, Pittsburgh, USA

**Keywords:** Concussion treatment, Rugby science, Rugby injury, MTBI

## Abstract

**Background:**

Sport-related concussion injuries are common in community Rugby Union. Despite research advancements in SRC management in recent years, little is known about the translation of research to practice within community Rugby Union cohorts.

**Aims:**

To develop and evaluate a novel system to track comprehensive SRC history, symptoms, and treatment data within community Rugby Union players.

**Methods:**

The Three-Phase Rugby Concussion System (TPRCS) collected comprehensive SRC data from players throughout the 2024-25 community Rugby Union season at baseline, 0–5 days post-SRC, and 0–5 days post-unrestricted return to play. Semi-structured interviews were conducted for stakeholders (players and club medics, *n* = 10) involved post-season, to evaluate participation experience, alongside perceived research facilitators, barriers, and usefulness.

**Results:**

Survey compliance ranged from 51% at baseline (*n* = 135), to 74% for post-SRC (*n* = 23), and post-clearance surveys (*n* = 23). The evaluation surveys received positive feedback for TPRCS’s ease of use and communication pathways, with player enthusiasm levels for study participation being the lowest-scoring survey response. The main facilitators for TPRCS included club culture, incentives, and personal contact, whilst the main barriers were absence of injury reporting and lack of prioritisation from players. Stakeholders identified education, injury treatment, and policy change as potential areas of usefulness.

**Conclusions:**

Medics and players in community Rugby Union are enthusiastic to progress practice and behaviours in line with research. Overall, this stakeholder-evaluated comprehensive SRC data collection system can serve as a novel SRC research framework across Rugby Unions.

**Supplementary Information:**

The online version contains supplementary material available at https://doi.org/10.1007/s11845-026-04485-x.

## Introduction

Sport-related concussion (SRC) management has evolved in recent years, shifting from complete rest to active recovery approaches and emphasising individualised rehabilitation targeting specific symptom and impairment domains [1]. The Concussion in Sport Group (CISG) recommends a stepwise, gradual return-to-play (RTP) protocol following SRC before returning to unrestricted sport participation, which takes a minimum of seven days to complete [2]. Use of exercise prescription post-concussion is supported by research, with the use of early exercise (within 2–14 days post-injury) shown to facilitate recovery and reduce the possibility of persisting concussive symptoms [3]. Some sporting bodies have developed their own RTP protocols, which include sport-specific guidance or drills, alongside extended periods of time out from play [4]. For example, the Irish Rugby Football Union (IRFU) mandates a 21-day sit-out period following an adult SRC, and a gradual RTP protocol [5].

Rugby Union (hereafter ‘rugby’) is among the sports with the highest SRC incidence rates [6], where the tackle event is the primary mechanism of injury [7]. These high SRC rates, coupled with a prior concussion being a risk factor for a subsequent concussion [8], render it imperative that SRC recovery and rehabilitation are undertaken with diligence to optimise player welfare. Despite this, research suggests that a full understanding of the severity of the injury, compliance with RTP protocols, and medical provision [9–11] is lacking in the community (amateur) game, where the largest population of players partake. Furthermore, little is known about pre- and post-injury risk factors of prolonged recovery in community players (e.g. medical history, SRC history, treatment provision, symptom presentation), or the management of players within the RTP period. An improved understanding of the medical history, presentation (i.e. symptoms), and management strategies implemented in community rugby players with SRC will enable clinicians to identify key areas that require attention in SRC care provision.

Thus, the research team developed and implemented the novel Three-Phase Rugby Concussion System (TPRCS) to comprehensively track medical history, symptoms, and treatment data within a community Irish Rugby Union population following SRC. We also employed stakeholder engagement research (SER), which is known to improve participant recruitment and retention, as well as knowledge dissemination [12–14], to ascertain facilitators and barriers to engagement and potential areas of usefulness. We hypothesised that potential positive research impacts, connection building, and prevalence of supportive environments would appear as potential facilitators, barriers, and areas of usefulness within this SRC data collection system’s evaluation, as has been found in prior non-sport-related concussion research [13].

## Methods

### Study design and participants

This study aimed to evaluate a comprehensive SRC data collection system within adult, community Irish Rugby Union players during the 2024-25 season. Teams involved in the All-Ireland League (AIL) who participated in ongoing injury surveillance using IRISweb, a bespoke online injury surveillance system [15], were recruited for this study. All study activities in the current study were in addition to their participation in existing Irish Rugby Injury Surveillance (IRIS). Recruitment occurred through the club’s designated injury recorder. In total, 14 teams were invited for involvement in this study, resulting in seven teams’ participation (five male and two female). As the AIL involves clubs playing at the highest amateur level across Ireland (the Republic of Ireland and Northern Ireland combined), representation from at least one club per Irish province was achieved to ensure balanced demographic representation. The project was approved by the University of Limerick research ethics committee (2016_06_19_EHS), in accordance with the Declaration of Helsinki.

### Procedures and data collection

#### TPRCS content development

**Fig. 1:**
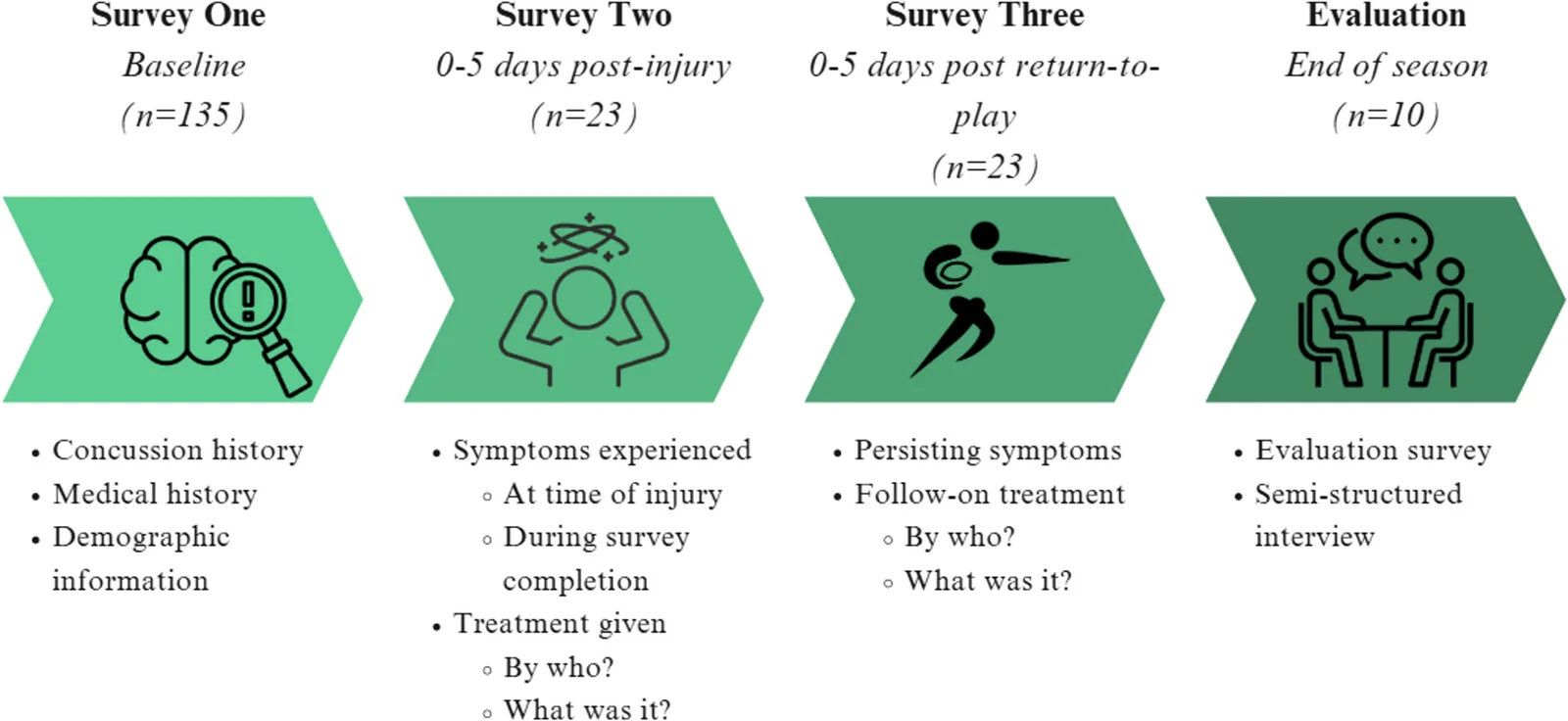
Procedures involved in data collection

Three surveys were developed by the research team, which includes expertise in SRC, neuropsychology, rugby-science, and statistics and data science (LP, IK, JM, HP, AK, MC, TC). The surveys contain elements of commonly used symptom inventories and consensus recommendations [2, 16], which offer content, expert, and face validity. All players were eligible for survey completion at baseline, whilst surveys Two and Three were distributed to players who sustained an SRC only. The contents of the three surveys are presented in Supplemental Material 1. Voluntary written informed consent was gathered from all participants within the study. The procedures involved in data collection are illustrated in Fig. 1.

##### Survey One

Survey One collected data on player demographic information (email, sex-specific sport category, age, club’s province), medical history (migraine, anxiety, depression, ADHD, car/motion sickness), and SRC history (number of diagnosed concussions, number of unreported/undiagnosed concussions, previous treatment recommendations, previous treatment providers). All players from participating clubs were invited to complete Survey One. Survey One’s dissemination occurred via an electronic link sent by the club’s injury recorder to the team’s normal electronic communication platform, alongside each injury recorder receiving a hard copy of the survey QR code, as generated by Qualtrics [17]. Each injury recorder was offered the option of an in-person visit from a member of the research team (LP) to discuss the study with players, with four clubs accepting the offer. The contents of the in-person visit are presented in Supplemental Material 2. Where in-person visits took place, the survey link was disseminated the same day, with a reminder message one week later.

##### Survey Two

Subsequent surveys applied only to players who sustained an SRC throughout the season. The research team was alerted to SRC events through injury recorder input to IRISweb, or direct communication with a member of the research team (LP). Post-concussion, Survey Two was distributed to players to be completed within 0–5 days post-injury. Survey Two investigated the signs and symptoms experienced immediately following the concussion, the setting and timing of concussion identification, the perceived percentage of feeling normal, any treatment advice given, and the treatment provider. Dissemination of Survey Two occurred through two pathways: where the injured player had completed Survey One, a member of the research team (LP) contacted the player through email once alerted to the event with the subsequent survey link, or the injury recorder sent a link to Survey One and Two to the player directly. If incomplete, a survey reminder was sent to the player on the last eligible day (day five post-injury).

##### Survey Three

Survey Three was eligible for players within 0–5 days of returning to unrestricted RTP. A member of the research team (LP) was informed of RTP through two pathways: player clearance on IRISweb or direct contact with the club’s injury recorder. Survey Three investigated time loss (in days) following the SRC, prolonged signs or symptoms, the player’s perceived percentage of back to feeling normal, any treatment given after the initial day of injury, and the treatment provider. This survey was emailed directly to the player once the research team was alerted to clearance. If incomplete, a survey reminder was sent to the player on the last eligible day (day five post-clearance).

#### Evaluation procedure

Within the final month of the 2024-25 season, all injury recorders involved, along with any players selected by them, were invited to participate in a semi-structured interview that included integrated survey questions. This process evaluated their experience of the survey study using Standards for Reporting Qualitative Research (SRQR) [18] and Consolidated Requirements for Qualitative Research (COREQ) [19]. The interviews were led by two moderators (LP, KB) who are involved in rugby in a player, and strength and conditioning capacity, respectively. One moderator had prior qualitative data experience (KB), and both were PhD researchers at the time of the interview. Some interviewees were known to the moderators through separate IRIS work; however, the research team does not believe that this familiarity impacted the content of the interviews. The interviews were held on Microsoft Teams and were recorded and transcribed. Interviewees provided verbal consent at the beginning of the interview, indicating their voluntary participation in the study. The interview schedule consisted of both open-ended and survey-style questions, as shown in Tables 1 and 2. The interview questions were based on previous literature within a rugby population [15, 20], adapted for use in relation to SRC injuries, and reviewed by experts within the research team. Interviewees provided insight into their experiences with TPRCS, including, but not limited to, possible facilitators, barriers, and the usefulness of the study results, which have previously been researched for other SRC tools [20, 21]. Follow-up queries from the moderators were intended to enhance the interviewer’s understanding of the stakeholder’s experience. Interviewees also completed a survey during the interview, where they rated elements of the TPRCS, such as ease of use, communication with the research team, and levels of player enthusiasm, on a 5-point Likert scale.

**Table 1:** Semi-structured interview script for Injury Recorder

Interview Introduction	Interviewer explains study design, rationale and describes the interview process. Give participant opportunity to introduce themselves and state what type of stakeholder they are within the team.	
Background Injury Recorder/Player	*How long have you been involved with the team (or other AIL teams) for*,* and in what capacity?* *Who does the team’s support staff consist of? Who is involved with concussion care of players?* *Have you been previously involved in concussion research studies? If so*,* what was the nature of the research*,* and was it also a survey study?*	
Injury Recorder and Player Survey Use		*Explain LIKERT scale 1–5* *1. Very poor* *2. Poor* *3. Neutral* *4. Good* *5. Very good* *Survey questions 1,*2*,*3*,*4*,*5* *Give participant opportunity to elaborate on each question if they wish*,* after each question*
Usefulness of Survey Study	*How do you feel the results of this survey study could be applied to be useful within a sporting setting (such as your rugby club)?* *If this survey study was to be implemented across more AIL teams*,* which survey from the list below do you feel is most important*,* and why?* *1) Concussion history* *2) Post-concussion treatment and symptoms* *3) Concussion clearance (treatment across the return-to-play period)*	
Barriers of Survey Study	*What would you feel are the main barriers to the implementation of this survey study*,* and why?* *Prompt if unclear; time taken to complete a task*,* interest or understanding of topic* *Do you have any thoughts on how to overcome these*,* or improve the study?*	
Facilitators of Survey Study	*What would you think are the main facilitators in the implementation of this survey study*,* and why?* *Prompt if unclear: What do you think are the main things that help make this survey study happen? Such as ease of access.*	
Final Question	*Is there anything else that you would like to share about your experience?*	

*Survey Questions

1. Please rate the ease of use of the IRIS Concussion Survey Study. LIKERT 1–5

a. Platform used for survey circulation. LIKERT 1–5

b. Communication pathways used. LIKERT 1–5

2. Please rate the usefulness of the IRIS Concussion Survey Study. LIKERT 1–5

3. Please rate the communication given from the research team throughout the study. LIKERT 1–5

4. Please rate enthusiasm levels for players to participate in this concussion study. LIKERT 1–5

5. Please rate how often the surveys were given. TOO OFTEN – JUST RIGHT – TOO INFREQUENT

**Table 2:** Semi-structured interview script for player

Interview Introduction	Interviewer explains study design, rationale and describes the interview process. Give participant opportunity to introduce themselves and state what type of stakeholder they are within the team.
Background Injury Recorder/Player	*How long have you played rugby for?* *What is the highest level that you have played at?* *What position do you play*,* usually?* *Have you been previously involved in concussion research studies? If so*,* what was the nature of the research and was it also a survey study?*
Injury Recorder and Player Survey Use	*Explain LIKERT scale 1–5* *1. Very poor* *2. Poor* *3. Neutral* *4. Good* *5. Very good* *Survey questions 1,*2*,*3*,*4*,*5* *Give participant opportunity to elaborate on each question if they wish*,* after each question*
Usefulness of Survey Study	*How do you feel the results of this survey study could be applied to be useful within a sporting setting (such as your rugby club)? This can be applicable to one specific survey*,* or all three surveys combined.* *If this survey study was to be implemented across more AIL teams*,* which survey do you feel is most important*,* and why?* *1) Concussion history* *2) Post-concussion treatment and symptoms* *3) Concussion clearance (treatment across the return-to-play period)*
Barriers of Survey Study	*What would you feel are the main barriers to the implementation of this survey study*,* and why?* *Prompt: such as time taken to complete a task*,* interest or understanding of a topic* *Do you have any thoughts on how to overcome these*,* or improve the study?* *Prompt: How could this process be easier for you as a player?*
Facilitators of Survey Study	*What would you think are the main facilitators in the implementation of this survey study*,* and why?* *Prompt if unclear: What do you think are the main things that help make this survey study happen? Such as ease of access*
Final Question	*Is there anything else that you would like to share about your experience?*

*Survey Questions

1. Please rate the ease of use of the IRIS Concussion Survey Study in relation to

a. Platform used for survey circulation. LIKERT 1–5

b. Communication pathways used. LIKERT 1–5

c. Time taken to complete the surveys. LIKERT 1–5

2. Please rate the usefulness of the IRIS Concussion Survey Study. LIKERT 1–5

3. Please rate the communication given from the research team throughout the study. LIKERT 1–5

4. Please rate your enthusiasm for participating in this concussion study. LIKERT 1–5

5. Please rate how often the surveys were given. TOO OFTEN – JUST RIGHT – TOO INFREQUENT

The use of two moderators and completion of an audit trail were used to enhance the trustworthiness of the data. Interviewees were not required to review transcripts to reduce the risk of bias within data collection [23]. There was no requirement for data saturation for this evaluation, as the prospect of new data emerging is likely as it continues to be collected [24]; therefore, interviews were conducted with all stakeholders willing to participate.

### Data analysis

For the qualitative analysis, transcripts were autogenerated by Microsoft Teams and manually audited by a member of the research team (LP). Nvivo 15 was used for coding by one researcher (LP) who conducted a deductive conventional content analysis due to the exploratory nature of the pilot study and analysis of social validation data [25, 26], rather than using themes to build an ‘*interpretive story’* [27]. This analysis was in line with the processes outlined by Elo and Kyngäs [28], where a categorisation matrix was formed and data were coded in line with categories. Similarities between codes were identified from the main categories of facilitators, barriers, and usefulness, to form generic and sub-categories, which co-authors had the opportunity to review. For the quantitative analysis, means and standard deviations (SD), and/or medians and interquartile ranges (IQR) were used to summarise the findings.

## Results

### Compliance with surveys

Survey One received 135 responses from 264 eligible participants (51.1% compliance). Surveys Two and Three received 23 responses out of a potential 31 SRCs (74.2% compliance).

### Evaluation

Injury recorders (male *n* = 3, female *n* = 2) and players (male *n* = 2, female *n* = 3), representing 6/7 (85.7%) of all involved clubs, participated in a semi-structured interview within the final month of the playing season. The demographic information is presented in Table 3.

**Table 3:** Demographic information of injury recorders and players evaluated

Injury Recorders		Players	
How long have you been involved in AIL rugby for?	10.1 (± 9.0) years	*How long have you played rugby for?*	9.8 (± 8.6) years
What type of stakeholder are you?	Physiotherapist/athletic therapist (*n* = 5)	*Highest level of rugby played?*	AIL (*n* = 4)
			Provincial division 1 (*n* = 1)
How many people within the team’s support staff? (i.e. coaches, medics)	7.2(± 1.1)	*What position?*	Winger (*n* = 2)
			Scrumhalf (*n* = 1)
			Back row (*n* = 1)
			Second row (*n* = 1)
Any previous involvement in concussion research?	Yes (*n* = 1)	*Any previous involvement in concussion research?*	Yes (*n* = 2)
	No (*n* = 4)		No (*n* = 3)

### Evaluation survey results

The results of the survey queries, presented in Likert scale format, from the interviews are shown in Table 4. For injury recorders, ‘very good’ was unanimous (5/5) for the ease of use of the survey study on communication pathways used, and the communication provided by the research team throughout the study. The frequency of survey distribution was ‘just right’ (4/5), with one injury recorder unsure at the time of the interview. All questions on the ease of use and usefulness received a minimum of a ‘good’ from all injury recorders. Injury recorders marked player enthusiasm for survey completion, ranging from poor (1/5) to very good (4/5). When queried which survey they felt to be the most important, the third survey (at time of clearance for return to play) was the most common response (5/5), followed by the first survey (4/5).

**Table 4:** Survey responses from injury recorders and player

Injury Recorders*	IR 1	IR 2	IR 3	IR 4	IR 5	Total mean (SD)	Total median (IQR)
Q1A) Ease of use of platform used for survey circulation	4	-	5	5	4	*4.5(0.6)*	*4.5(1)*
Q1B) Ease of use of communication pathways used	5	5	5	5	5	*5(0)*	*5(0)*
Q2) Usefulness of study	4	5	5	5	4	*4.6(0.6)*	*5(1)*
Q3) Communication given from research team	5	5	5	5	5	*5(0)*	*5(0)*
Q4) Player enthusiasm levels	4	3	2	3	4	*3.2(0.8)*	*3(1)*
Q5) Frequency of surveys	JR	JR	JR	JR	-	*-*	*-*
Q6) Which survey is most important?	1, 3	1, 2, 3	1, 3	3	1,2,3	*-*	*-*
Players	***P1***	***P2***	***P3***	***P4***	***P5***	***Total mean (SD)***	***Median (IQR)***
Q1A) Ease of use of platform used for survey circulation	5	5	4	5	4	4.6(0.6)	5(1)
Q1B) Communication pathways used	5	5	3	5	4	4.4(0.9)	5(1)
Q1C) Time taken to complete surveys	5	5	4	5	5	4.8(0.5)	5(0)
Q2) Usefulness of study	5	5	5	5	5	5(0)	5(0)
Q3) Communication given from research team	5	5	4	5	4	4.6(0.6)	5(1)
Q4) Enthusiasm levels	5	5	3	5	4	4.4(0.9)	5(1)
Q5) Frequency of surveys	JR	JR	TI	JR	JR	-	-
Q6) Which survey is most important?	1, 3	3	3	1, 2, 3	3	-	-

*Q1 – Q5 answered on a LIKERT 1–5 scale (1 very poor, 5 very good), Q5 answered too infrequent (TI), just right (JR), or too frequent (TF)

For players, ‘very good’ was unanimous (5/5) for the usefulness of the survey study. The ease of use of the platforms, the time taken to complete surveys, and the communication provided by the research team all scored a minimum of ‘good’ by all players (5/5). Communication pathways used and enthusiasm for participation in the study ranged from ‘neutral’ (1/5) to a minimum of ‘good’ (4/5). The frequency of surveys ranged from ‘too infrequent’ (1/5) to ‘just right’ (4/5). When queried which survey they felt to be the most important, Survey Three was the most common response (5/5). Players, similar to injury recorders, felt that it reinstilled readiness to RTP, rather than just the mandatory sit-out period finish date.

### Facilitators, barriers, and usefulness

**Fig. 2:**
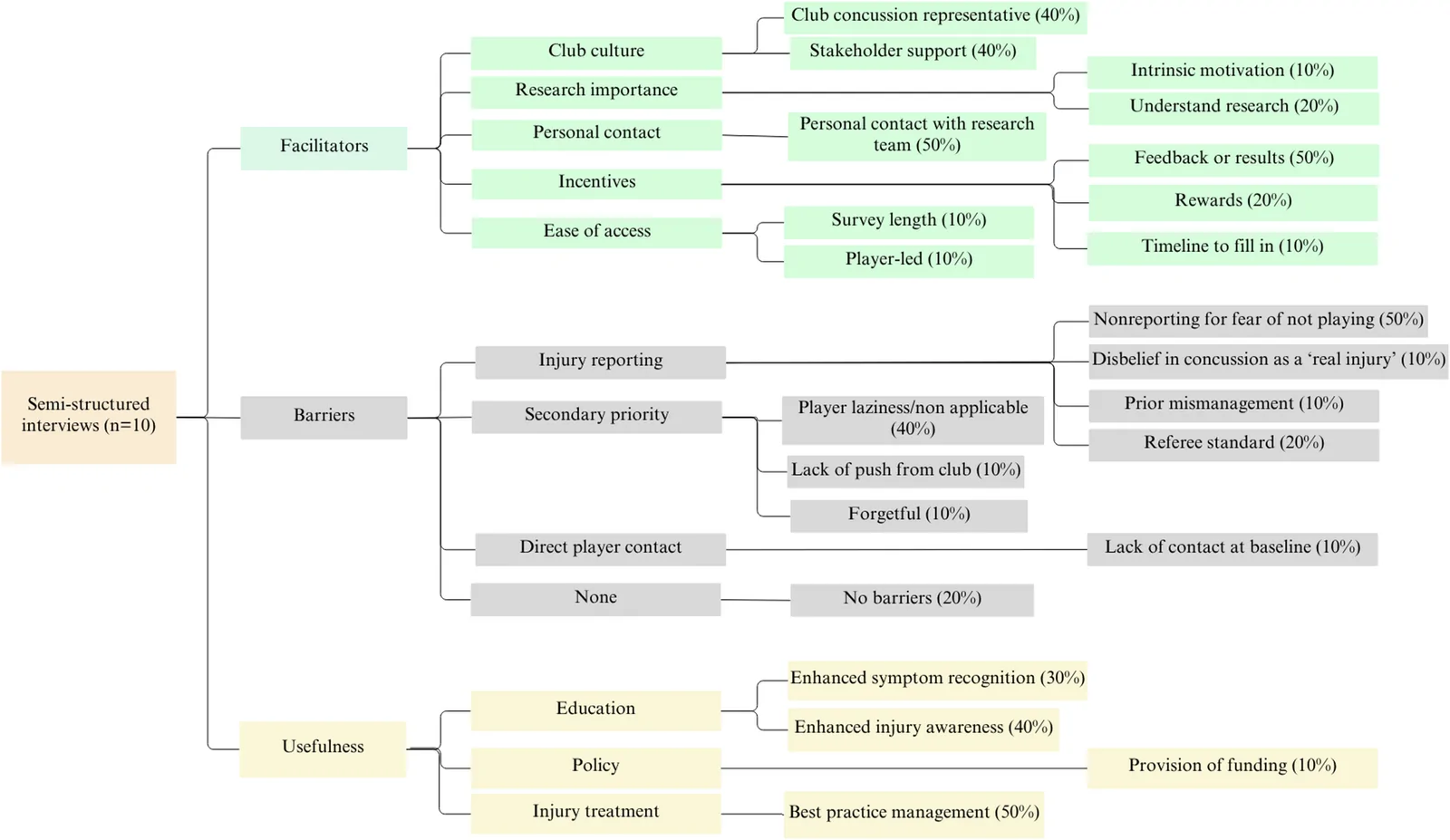
Facilitators, Barriers, and Usefulness of the comprehensive SRC survey

Three main categories were derived from the qualitative evaluation analysis: facilitators, barriers, and usefulness. An overview of this is presented in Fig. 2.

#### Facilitators

##### Club culture

Elements of club culture were discussed in relation to club concussion representatives (*n* = 4) and stakeholder support (*n* = 4). In terms of having a concussion representative in the club to manage the injury, this was drawn upon by those who currently hold this role within their club, as well as others who would support its implementation. Participants felt that the inclusion of a concussion representative role would drive compliance with the research. Stakeholder support was also discussed in terms of their influence on concussion culture in clubs and their effects on research compliance. Stakeholders involved in the discussion by both players and injury recorders included alickadoos, the head coach, the team captain, and teammates. Their roles were emphasised as having a potential influence on concussion culture in the club, regarding perception of injury, advocacy for concussion awareness, and influence on participation and/or compliance with concussion research. For example,

***P3*** *’I think a really important factor would be the*,* suppose*,* like a driving force from each club*,* like the S&C we have at our club this year was kind of big on*,* on holding people accountable for it. So it’s kind of*,* I suppose the idea of kind of like just reinforcing how important it is and like the*,* from both a player protection side and kind of like an informing side.’*

***IR4*** *’…it will be a trickle-down effect*,* if the top people in the club are taking more notice of it than the players*,* then there has to be an effect on the players. You know and if*,* if it’s the club that is driving us*,* the club*,* the players have to take responsibility in*,* in being more aware about and more proactive about’*.

##### Research importance

Player perceptions of research importance as a facilitator were discussed in relation to intrinsic motivation (*n* = 1) and understanding research (*n* = 2). One participant discussed their intrinsic motivation, noting that their current studies in a sport-related undergraduate degree were the primary facilitator of their participation. The role of understanding research was discussed in relation to understanding the ‘why’ behind the research, which drives compliance. Due to the community population in this study, one participant discussed the potential lack of understanding among players regarding the day-to-day implications of SRC (outside of sport) and suggested that education on these topics may drive compliance in the study. For example,

***P4*** *’I guess more like transparency or visibility behind the research*,* because for us it was*,* there was less information about what the research project was and it was just like*,* here’s a research project going on*,* would love your input on the survey. So maybe more kind of toolkits or communication to give to coaches to then share with players about the why behind the project and then that might like motivate people to participate.’*

##### Personal Contact 

Personal contact with the research team was discussed by five interviewees. All participants felt that personal contact, whether in person or through email, facilitates survey studies such as this, as it creates a more relatable and personable research approach through direct interaction. Two injury recorders also discussed the benefits of the research team sending reminder texts once they were alerted to an SRC, as it reminded them to disseminate the survey and allow for follow-up and accountability. For example,

***IR1*** *: ’And for our lads, generally, if you’re not directly in front of them, they don’t really take notice of it.’*

***IR5*** *: ‘…you came out to our guys and you spent time with them and explain things, and that was quite impactful I think as opposed to not getting an e-mail or me texting to the group’*

##### Incentives

Feedback and results (*n* = 5), rewards (*n* = 2), and the timeline for completing the surveys (*n* = 1) were discussed as possible incentives to drive compliance with the study. Feedback or result provision was thought to drive compliance, as it would make the research more personal to the cohort involved, raise awareness of the injury, or foster a desire to utilise feedback to improve systems. Rewards were discussed in relation to both the injury recorders involved to incentivise their involvement, such as continued professional development or merchandise, alongside rewards within teams, such as a raffle, may facilitate future involvement. For example,

***IR3*** *‘People like getting some free stuff sometimes. So it incentivises them and you know you*,* you probably will see buy in from someone a lot more than if they were just sitting down giving up their own time.’*

##### Ease of access

Survey length (*n* = 2) and player-led (*n* = 1) were discussed as facilitators for this survey study. Two interviewees emphasised the importance of facilitating surveys that do not take too much time as being key to survey compliance. One interviewee, an injury recorder, discussed the study design of the surveys being player-led as a facilitator in their compliance throughout the season, as it mitigates the potential burden they may have. For example,

***IR1:*** *‘…it’s managed by them and if I need to give the odd message to sort of encourage them to complete it, that’s no problem like this, there’s no time out. But it’s nice having one that’s player led.’*

#### Barriers

##### Injury reporting

The initial reporting of the injury was discussed in terms of nonreporting in fear of not playing (*n* = 5), disbelief in concussion as a real injury (*n* = 1), prior mismanagement (*n* = 1), and referee standards (*n* = 2). Nonreporting of the injury was acknowledged as a significant barrier, as it limits the capacity to identify the injury and thereby distribute surveys. For example,

***P1*** *‘…maybe some of the players might think*,* oh*,* if I do*,* then I won’t be able to play and they might not be considering their own welfare as they should in that’*.

***IR2*** *‘So obviously the players never want to be injured*,* and concussion is like a swear word to them because they know they’re getting a certain period out.’*

##### Secondary priority

A lack of prioritisation was discussed as a barrier to survey compliance, such as player laziness or the feeling that the injury was inapplicable to players (*n* = 4), alongside a lack of push from the club (*n* = 1), and forgetfulness (*n* = 1). Some injury recorders and players believed that concussion is not a top priority for players, as there is a lack of personal connection or experience in the injury, which is further facilitated by the fact that the club may not be advocating for the injury, or whereby players may be forgetful after an initial survey link is distributed and require a reminder. For example,

***IR1*** *‘I think it’s*,* it’s getting player buy in and not necessarily because they don’t think that the injury reporting or concussion monitoring is a good idea*,* it’s just that they don’t see how it applies to them.’*

***P2*** *‘…I think it just people are busy and they*,* you know*,* selfish as well. Like you know*,* they’re like doesn’t really apply to me I don’t need to do it.’*

##### Direct player contact

One player mentioned that the lack of direct communication with all eligible players during pre-season was a potential barrier, as this study design only allowed for player contact after they had completed the initial survey.

**P4: ‘** *I would say probably for you guys not having a direct line of communication to everyone participating might be a bit of a barrier or relying on clubs to properly identify or highlight people who have had concussion and then making sure they partake in it.’*

##### None

One player felt that there were no barriers to participation. For example,

***P5*** *’I didn’t feel like there was any barriers*,* in my opinion now*,* I feel like they were quite easy to get through*,* questions were quite specific and like it wasn’t really hard to fill out.’*

#### Usefulness

##### Education

Enhanced symptom recognition (*n* = 3) and enhanced injury awareness (*n* = 4) were identified as areas where the survey could prove useful. Two players and an injury recorder discussed the heightened awareness of concussion symptoms that resulted from their participation in the study. Two players and two injury recorders also discussed the potential for study results to upskill players and club stakeholders alike to educate themselves on proper management and awareness of potential consequences if mismanaged. For example,

***IR4*** *‘…it got lads a little bit more engaged in the importance of it and like in the past*,* it would have been aw you do*,* like they just took it as got a head knock*,* do a couple of exams*,* that’s it*,* I’m back in three weeks. but it’s sort of*,* it got them a little bit more conscious of the importance of it.’*

***P4*** *‘… I think it would probably help people to identify what symptoms were related to concussion like*,* say*,* if they got mild concussion and weren’t like*,* it wasn’t totally apparent that they’d had it. So tracking kind of trends in the symptoms of peoples like own experience might help to map that*,* and then have coaches and players better understand when it really is related to concussion and then you can protect yourself a bit in that’*.

##### Injury treatment

The potential for results to be used to inform best practice management, such as concussion prehabilitation or management plans (*n* = 3), improved diagnostics (*n* = 1), and increased medic accountability (*n* = 1) were discussed for survey usefulness, both from the results of the survey and the feedback from the evaluation. For example,

***IR1*** *‘For me it would just be interesting to see what the player feedback is and what they feel is needed or where we could be better at implementing rehab ideas.’*

***IR5*** *‘…like I come across just like really wide range of knowledge and a really wide range of ‘best practice’ being applied across teams.’*

##### Policy

One injury recorder discussed the potential for survey results to inform policy and allow for enhanced funding to aid concussion care within the domestic game. For example,

***IR2*** *‘I think it will be like almost a cascade if there’s better funding to get more access to medical staff*,* then you’ll get more buy in from the girls that will result in better*,* like in a stronger squad*,* better quality of play like I just think it will cascade down’*.

It should be noted that […] in the above refers to elisions where extraneous words have been excluded without altering the meaning of the quotation.

## Discussion

This is the first mixed-methods study within community Irish Rugby Union players investigating medical history, symptoms, and treatments following SRC. Furthermore, this is the first investigation into the experience of participation for community rugby stakeholders, despite the growing focus on SRC research. Our results supported the development and implementation of the Three-Phase Rugby Concussion System (TPRCS), highlighting evaluation metrics for the implementation procedures, as well as insightful barriers, facilitators, and perceptions of usefulness from stakeholders involved. The evaluation component identified a positive response from both injury recorders and players, in terms of platform ease of use, survey dissemination techniques and frequency, and the belief in the TPRCS’s usefulness within this cohort. A less prominent aspect was players’ enthusiasm to participate in the study. However, compliance rates by all players for Survey One (51%) and the subsequent post-SRC surveys (74%) contradict this finding, likely highlighting strengths in areas of data collection processes, where, despite a lack of enthusiasm, action was taken. Altogether, this stakeholder-evaluated comprehensive SRC data collection procedure may serve as a framework for SRC research in other Rugby Unions.

### Facilitators and barriers

The facilitators and barriers identified in this study align with those discovered in other areas of SRC qualitative research, such as baseline testing or SRC reporting [22, 29, 30]. Whilst this study was a niche undertaking in terms of content, study design, and timepoints for dissemination, the facilitators and barriers, in part, align with those of Salmon et al. (2024) [22] who investigated stakeholders’ perceptions of baseline testing with concussion management in the New Zealand community game. Although the outcomes differ between studies, facilitators and barriers remain similar, such as stakeholder support and team culture, whereby stakeholders within the team promoted testing, facilitating the study rollout [22]. The direct involvement of stakeholders (e.g. injury recorders) within the current study may act as a potential rationale for high levels of compliance throughout the season. This combats low player enthusiasm, which may be rooted in the principles of social validation, where individuals rely on others to decide what actions to take [26, 31]. However, any future roll-out should be cognisant of the influence of other stakeholders, such as coaches and players, on research participation as well.

Social validation may also underpin personal interaction with the research team being one of the most frequently referenced facilitator categories, mentioned by half of the interviewees. A member of the research team speaking directly to participants was deemed positively influential by those who experienced it, and requested by those where the club did not opt for an in-person visit, or by players who were absent during the visit. Face-to-face interactions have been previously noted in literature to increase compliance to a task, potentially due to the use of nonverbal cues, development of empathy, and trust between the researcher and participant [32, 33].

Feedback and results were also discussed by half of the interviewees as a facilitator, with a desire for the participant’s input to be used practically to help inform stakeholders about the reality of SRC treatment in community rugby. These results may also act as a potential protective mechanism to the barrier of players’ feeling as though the study does not apply to them or placing secondary importance on the task. The desire for research to be relevant to participants involved has been previously found in concussion research [13, 34]. This facilitator may juxtapose the barrier of player laziness, often due to the feeling that the information does not apply to participants, which was discussed by 40% of interviewees.

Other barriers disclosed mainly focused on external variables, such as issues with injury reporting and secondary prioritisation. Issues with injury reporting due to a myriad of reasons, such as fear of not playing and disbelief in concussion as a ‘real injury’, have been previously documented in rugby literature [35, 36]. However, the standard of refereeing impacting SRC reporting may be a novel characteristic in community sport, whereby two participants had negative experiences of SRC misdiagnoses by referees as a result of proactive ‘recognise and remove’ campaigns [37]. These findings highlight the consistently evident issue of non-reporting within the community game and the potential knock-on effect this may have on SRC research.

### Usefulness

The usefulness of TPRCS findings was discussed by participants in both process and outcome measures. Process-driven outcomes focused on SRC education, with interviewees noting increased knowledge of concussion symptoms and heightened injury awareness through survey participation. This is in line with prior SRC research where increased knowledge occurred after conducting baseline testing research [34], and where a player has previously sustained an SRC injury [38]. Overall, the research team found that both players and injury recorders were enthusiastic about the prospect of further education, with some injury recorders suggesting outcomes be reported back to wider club stakeholders to enhance SRC education in a broader sense. Prior SRC studies have consistently concluded that both a desire for further education and its necessity exist [30, 39, 40]; however, despite advancements in recent years, it appears there may be a gap between research completion and knowledge transfer to stakeholders [41]. Meanwhile, usefulness in terms of best practice management demonstrates enthusiasm and a desire among those involved to advance SRC treatment and management in accordance with current best practices.

### Clinical implications

Stakeholders in community Rugby Union are eager to progress SRC management procedures and enhance knowledge of the injury. Facilitators and barriers should be accounted for within future protocol development or implementation of comprehensive SRC data collection within this cohort. Stakeholder engagement research should be considered in policy development by respective Rugby Unions to ensure germane policy creation and optimisation of compliance.

### Limitation

This study includes possible limitations to its generalisability; firstly, volunteer bias may be evident in those who chose to participate in the study’s evaluation component. The research was also implemented on a pilot study basis, allowing for evaluation before a wider study rollout. Consequently, a small sample size was used within the community Rugby Union cohort.

## Conclusion

Stakeholders in community Irish Rugby Union are generally keen to participate in SRC research to enhance both education and practice in SRC knowledge and injury management. The TPRCS was highly regarded among injury recorders and players, with certain aspects, such as personal interaction with the research team, feedback and results, and a positive concussion culture within the club, being frequently flagged as facilitators. Barriers remain, similar to other SRC research, such as issues with injury reporting and players not prioritising research or feeling that the data are irrelevant to them. The successful implementation of SRC research within community Rugby Union is crucial to allow for a well-rounded knowledge and understanding of ground-level SRC injuries, to inform stakeholders involved, and promote best practices within the cohort.

## Supplementary Information

Below is the link to the electronic supplementary material.

Supplementary Material 1 (DOCX 52.8 KB)

## Data Availability

All data from the development and evaluation procedures have been supplied.
