# Supplementary material for: The development and mixed-method stakeholder evaluation of a sport-related concussion symptom and treatment monitoring system in community rugby
Source: Ir J Med Sci. 2026 Jun 19;195(5):3149–60. doi: 10.1007/s11845-026-04485-x (PMC13739973; doi:10.1007/s11845-026-04485-x)
Supplement: Supplementary file 1 [file 11845_2026_4485_MOESM1_ESM.docx]

**Supplemental Material 1; Surveys developed**

**Survey One**

1.	What sex-specific sport category do you participate in in AIL Rugby in Ireland?

- Male
- Female

2. What age are you?

________________________________________________________________

3.	What Irish province is your club located in?

- Leinster
- Munster
- Connacht
- Ulster

4. Do you have a history of the following (select all that apply)?

- Migraine headaches
- Car/motion sickness
- Anxiety
- Depression
- ADHD

5. Have you ever had a head injury that was diagnosed by a healthcare provider as a concussion (not inclusive of any concussions sustained in the past 5 days)?

- Yes
- No

*Display This Question:*

*If Have you ever had a head injury that was diagnosed by a healthcare provider as a concussion (not... = Yes*

6. How many concussions have you had in your lifetime?

- 1
- 2
- 3
- 4
- 5
- 6
- 7
- 8+

*Display This Question:*

*If Have you ever had a head injury that was diagnosed by a healthcare provider as a concussion (not... = Yes*

7. How many of your previous diagnosed concussions resulted in you losing consciousness (being knocked out)?

- 0
- 1
- 2
- 3
- 4
- 5
- 6
- 7
- 8+

*Display This Question:*

*If Have you ever had a head injury that was diagnosed by a healthcare provider as a concussion (not... = Yes*

8. How many of your diagnosed concussions occurred during sport (e.g., football, rugby, hurling/camogie, Gaelic football, etc.)?

- 0
- 1
- 2
- 3
- 4
- 5
- 6
- 7
- 8+

*Display This Question:*

*If Have you ever had a head injury that was diagnosed by a healthcare provider as a concussion (not... = Yes*

9. When was your most recent diagnosed concussion?

- During the past year
- Between 1 and 3 years ago
- More than 3 years ago

10. How many concussions have you had while playing rugby that you did not report or were not diagnosed by a physio, team doctor, or other healthcare provider?

- 0
- 1
- 2
- 3
- 4
- 5
- 6
- 7
- 8+

11. Did you receive any treatment recommendations following your most recent concussion (not inclusive of any concussions sustained in the past 5 days)?

- Yes
- No

*Display This Question:*

*If Did you receive any treatment recommendations following your most recent concussion (not inclusiv... = Yes*

12. From whom did you receive treatment recommendations following your most recent concussion (not inclusive of any concussions sustained in the past 5 days - check all that apply)?

- Physiotherapist/Athletic Therapist/Sport Therapist
- Team Doctor
- General Practitioner (GP)
- Pediatrician
- Neurologist
- Other

*Display This Question:*

*If Did you receive any treatment recommendations following your most recent concussion (not inclusiv... = Yes*

13. Which of the following treatment recommendations did you receive following your most recent concussion (not inclusive of any concussions sustained in the past 5 days)?

- Physical Rest (NO exercise, resistance training, etc.)
- Cognitive Rest (NO mobile phones, computer, video games etc.)
- School or Work Accommodations (missed school/work, extra time, assistance, modifications, etc.)
- Physical Activity (walking, exercise, resistance training, etc.)
- IRFU Gradual Return-to-Play Protocol
- Sleep Recommendations
- Physiotherapy
- Medications
- Other

**Survey 2; Post-Concussion Survey**

1. How many days has it been since you suffered a concussion?

- 0 (same day)
- 1
- 2
- 3
- 4
- 5

2. Which of the following signs and symptoms did you experience immediately following your concussion?

- Loss of consciousness (knocked out)
- Difficulty remembering things before the injury
- Difficulty remembering things after the injury
- Confusion or disorientation
- Dizziness
- Vomiting
- Balance problems
- Nausea
- Vision problems
- Headache
- Neck pain
- Difficulty concentrating
- Feeling foggy

3. Did your concussion occur during practice/training or a match?

- Practice/training
- Match

4. When was your concussion reported or identified?

- Immediately
- Later during the same practice/training or match
- Later that day after practice/training or the match
- The following day
- 2 or more days later

5. Which of the following signs and symptoms are you currently experiencing?

- Difficulty remembering
- Dizziness
- Balance problems
- Sadness
- Nausea
- Vision problems
- Headache
- Difficulty concentrating
- Nervousness
- Feeling foggy
- Sleep problems
- Neck pain
- None

6. How would you rate your percent (%) back to normal right now? 0% indicates no improvement, where 100% indicates fully recovered.

|  | 0 | 10 | 20 | 30 | 40 | 50 | 60 | 70 | 80 | 90 | 100 |
| --- | --- | --- | --- | --- | --- | --- | --- | --- | --- | --- | --- |

| - % back to normal () | -  |
| --- | --- |

7. Did you receive any treatment recommendations following your concussion, on the day of your initial injury?

- Yes
- No

*Display This Question:*

*If Did you receive any treatment recommendations following your concussion, on the day of your initi... = Yes*

8. From whom did you receive follow-on treatment recommendations following your concussion on the day of your initial injury (check all that apply)?

- Physiotherapist/Athletic Therapist/Sport Therapist
- Team Doctor
- General Practitioner (GP)
- Pediatrician
- Neurologist
- Other

*Display This Question:*

*If Did you receive any treatment recommendations following your concussion, on the day of your initi... = Yes*

9. Which of the following treatment recommendations did you receive following your concussion, on the day of your initial injury (select all that apply)?

- Physical Rest (NO exercise, resistance training, etc.)
- Cognitive Rest (NO mobile phone, computer, video games, etc.)
- School or Work Accommodations (missed school/work, extra time, assistance, modifications, etc.)
- Physical Activity (walking, exercise, resistance training, etc.)
- IRFU Gradual Return-to-Play Protocol
- Sleep Recommendations
- Physiotherapy
- Medications
- Other

**Survey Three; Concussion Clearance Survey**

1. Are you completing this survey after having been cleared to unrestricted return-to-play? If not, please exit and complete once clearance has been given.

- Yes
- No

*Display This Question:*

*If Are you completing this survey after having been cleared to unrestricted return-to-play? If not,... = Yes*

2. How many days has it been since you suffered a concussion?

________________________________________________________________

*Display This Question:*

*If Are you completing this survey after having been cleared to unrestricted return-to-play? If not,... = Yes*

3. If you are still suffering from concussive symptoms, which of the following are you currently experiencing (select all that may apply)?

- Difficulty remembering
- Dizziness
- Balance problems
- Sadness
- Nausea
- Vision problems
- Headache
- Difficulty concentrating
- Nervousness
- Feeling foggy
- Sleep problems
- Neck pain
- None

*Display This Question:*

*If Are you completing this survey after having been cleared to unrestricted return-to-play? If not,... = Yes*

4. How would you rate your percent (%) back to normal right now? 0% indicates no improvement, where 100% indicates fully recovered.

|  | 0 | 10 | 20 | 30 | 40 | 50 | 60 | 70 | 80 | 90 | 100 |
| --- | --- | --- | --- | --- | --- | --- | --- | --- | --- | --- | --- |

| - % back to normal () | -  |
| --- | --- |

*Display This Question:*

*If Are you completing this survey after having been cleared to unrestricted return-to-play? If not,... = Yes*

5. Did you receive any treatment recommendations following your concussion, after the day of your initial injury?

- Yes
- No

*Display This Question:*

*If Are you completing this survey after having been cleared to unrestricted return-to-play? If not,... = Yes*

*And Did you receive any treatment recommendations following your concussion, after the day of your in... = Yes*

6. From whom did you receive follow-on treatment recommendations following your concussion after the day of your initial injury (check all that apply)?

- Physiotherapist/Athletic Therapist/Sport Therapist
- Team Doctor
- General Practitioner (GP)
- Pediatrician
- Neurologist
- Other

*Display This Question:*

*If Are you completing this survey after having been cleared to unrestricted return-to-play? If not,... = Yes*

*And Did you receive any treatment recommendations following your concussion, after the day of your in... = Yes*

7. Which of the following treatment recommendations did you receive following your concussion, after the day of your injury (select all that apply)?

- Physical Rest (NO exercise, resistance training, etc.)
- Cognitive Rest (NO mobile phone, computer, video games, etc.)
- School or Work Accommodations (missed school/work, extra time, assistance, modifications, etc.)
- Physical Activity (walking, exercise, resistance training, etc.)
- IRFU Gradual Return-to-Play Protocol
- Sleep Recommendations
- Physiotherapy
- Medications
- Other

**Supplemental Material 2 – Content of Club Visit**

**Introduce:** Firstly, thanks very much to you all for giving me a few minutes of your time, and for your coach and physio for helping to facilitate a suitable time for it to happen. My name is Laura I am a PhD researcher in University of Limerick with the Irish Rugby Injury Surveillance Project (IRIS). Some of you may be more familiar with IRIS than others, but just as a brief overview it is an injury surveillance system formed between University of Limerick and the IRFU* which allows for annual report production of injuries in the AIL**; what injury was most prevalent, what caused the most amount of days missed etc. If anybody has interest in it the reports are freely available online. I’m here to discuss an upcoming concussion study as part of this project.

**Concussion Research**: My specific focus as part of the IRIS team is on concussion in rugby in Ireland. There are a lot of exciting progressions in rugby research, some which you may be more acquainted with than others such as new technology mouthguards and new tackle height trial (sternum level and below only). We wish to add to the body on ongoing concussion in rugby research specifically within Ireland by creating the opportunity for data collection and solid evidence gathering of Irish amateur Rugby cohorts (AIL teams) to figure out what’s happening on the ground in Ireland with regards to concussion symptoms and treatments specifically. Concussions are highly individual injuries, similar to the understanding that not all knee injuries are the same (be it an ACL, meniscus etc.), concussions are different for each person. Your symptoms may actually dictate what treatment you should optimally receive or what rehabilitation route you may go down following on from a concussion injury. So this is a huge opportunity to get data on AIL rugby specifically so we can highlight areas which may be beneficial to impact policy and enhance player welfare.

**Study Design**: The research team and I hope to achieve the initial stages of this through surveys to yourselves (the players), consisting of 1 or 3 surveys, with all results confidential and unidentifiable. Each player is given a pseudonym so no data will ever be tracked back to players, and it is solely to be used for research purposes. This is a pilot study featuring 6 male teams and between 2-4 female teams, with representation from across Ireland, with at least 1 club per province represented. Anybody within the first team squad can fill in the initial survey (approx. 10 mins) which consists of some concussion history (did you get treatment, is so by who and what) and brief medical history of migraine disorder, ADHD, anxiety etc. Whether you have had a concussion or not, all information is so important to gather to give a true depiction of concussion history and prevalence in AIL squads.

If somebody gets concussed there are 2 more surveys, one within 5 days of injury and one within 5 days of clearance from injury. These focus on symptoms felt, recording treatment given etc. The idea is then at the end of the year we can analyse results to see an array of things such as if any baseline information was linked to increased susceptibility of sustaining a concussion, any particular symptoms were linked to a longer concussion recovery or what treatment might be correlated to a quicker or slower recovery.

The idea of this is to better understand concussion within this very specific population so that clinicians can be better informed when dealing with Irish amateur rugby players as to how to best manage players, what treatments to use and recovery duration expectation. Ultimately, helping to improve player welfare. It’s a huge opportunity to get data to help this specific cohort of players both presently and in the future of AIL.

The initial survey will be sent out by your physio or coach via WhatsApp or the platform the team uses for communication, and I would appreciate if everybody had 10 minutes to spare to fill it out, it will really add to our concussion understanding. My contact information is available for anybody with queries further on down the line through your coach, injury recorder/physio or on the QR codes available on the sheet available within your first aid room.

Are there any questions or queries?

* Irish Rugby Football Union

**All-Ireland League

**Supplementary Material 3: Qualitative Checklists**

**Standards for Reporting Qualitative Research (SRQR): A Synthesis of Recommendations**

| **Item No.** | **SRQR Item** | **Addressed in Manuscript? (Yes/No)** | **Location (Page/Section)** |
| --- | --- | --- | --- |
| 1 | Title and abstract | Yes | Title page |
| 2 | Abstract | Yes | Abstract |
| 3 | Problem formulation | Yes | Introduction |
| 4 | Purpose or research question | Yes | Introduction |
| 5 | Qualitative approach and research paradigm | Yes | Methods |
| 6 | Researcher characteristics and reflexivity | Yes | Methods |
| 7 | Context | Yes | Methods |
| 8 | Sampling strategy | Yes | Methods |
| 9 | Ethical issues pertaining to human subjects | Yes | Methods |
| 10 | Data collection methods | Yes | Methods |
| 11 | Data collection instruments and technologies | Yes | Methods |
| 12 | Units of study | Yes | Methods |
| 13 | Data processing | Yes | Methods |
| 14 | Data analysis | Yes | Methods |
| 15 | Techniques to enhance trustworthiness | Yes | Methods |
| 16 | Synthesis and interpretation | Yes | Results |
| 17 | Links to empirical data | Yes | Results |
| 18 | Integration with prior work, implications, transferability, and contribution(s) to the field | Yes | Discussion |
| 19 | Limitations | Yes | Discussion |
| 20 | Conflicts of interest | Yes | Conflicts of interest |
| 21 | Funding | Yes | Conflicts of interest |

**Consolidated Criteria for Reporting Qualitative Research (COREQ)**

| **Item No.** | **Domain** | **COREQ Item Description** | **Addressed? (Yes/No)** | **Location (Page/Section)** |
| --- | --- | --- | --- | --- |
| **Domain 1: Research team and reflexivity** |  |  |  |  |
| 1 | Interviewer/facilitator | Which author/s conducted the interview or focus group? | Yes | Methods |
| 2 | Credentials | What were the researcher's credentials? | Yes | Title Page |
| 3 | Occupation | What was their occupation at the time of the study? | Yes | Methods |
| 4 | Gender | Was the researcher male or female? | Yes | Methods |
| 5 | Experience and training | What experience or training did the researcher have? | Yes | Methods |
| 6 | Relationship established | Was a relationship established prior to study commencement? | Yes | Methods |
| 7 | Participant knowledge of the interviewer | What did the participants know about the researcher? | Yes | Table 1 and 2 |
| 8 | Interviewer characteristics | What characteristics were reported about the interviewer/facilitator? | Yes | Methods |
| **Domain 2: Study design** |  |  |  |  |
| 9 | Theoretical framework | What methodological orientation was stated to underpin the study? | Yes | Methods |
| 10 | Sampling | How were participants selected? | Yes | Methods |
| 11 | Method of approach | How were participants approached? | Yes | Methods |
| 12 | Sample size | How many participants were in the study? | Yes | Methods |
| 13 | Non-participation | How many people refused to participate or dropped out? | Yes | Methods |
| 14 | Setting | Where was the data collected? | Yes | Methods |
| 15 | Presence of non-participants | Was anyone else present besides the participants and researchers? | N/A |  |
| 16 | Description of sample | What are the important characteristics of the sample? | Yes | Table 3 |
| 17 | Interview guide | Were questions, prompts, guides provided by the authors? | Yes | Table 1 and 2 |
| 18 | Repeat interviews | Were repeat interviews carried out? | N/A |  |
| 19 | Audio/visual recording | Did the research use audio or visual recording? | Yes | Methods |
| 20 | Field notes | Were field notes made during/after the interview? | No |  |
| 21 | Duration | What was the duration of the interviews or focus groups? | No |  |
| 22 | Data saturation | Was data saturation discussed? | Yes | Methods |
| 23 | Transcripts returned | Were transcripts returned to participants for comment or correction? | No | Methods |
| **Domain 3: Analysis and findings** |  |  |  |  |
| 24 | Number of data coders | How many data coders coded the data? | Yes | Methods |
| 25 | Description of the coding tree | Did authors describe the coding tree? | Yes | Methods |
| 26 | Derivation of themes | Were themes identified in advance or derived from the data? | N/A |  |
| 27 | Software | What software, if applicable, was used to manage the data? | Yes | Methods |
| 28 | Participant checking | Did participants provide feedback on the findings? | Yes | Methods |
| 29 | Quotations presented | Were participant quotations presented to illustrate the themes? | Yes | Results |
| 30 | Data and findings consistent | Was there consistency between the data presented and the findings? | Yes | Results |
| 31 | Clarity of major themes | Were major themes clearly presented in the findings? | Yes | Results |
| 32 | Clarity of minor themes | Is there a description of diverse cases or minor themes? | Yes | Results |
